# Traditional Uses, Phytochemistry, and Bioactivities of *Cananga odorata* (Ylang-Ylang)

**DOI:** 10.1155/2015/896314

**Published:** 2015-07-30

**Authors:** Loh Teng Hern Tan, Learn Han Lee, Wai Fong Yin, Chim Kei Chan, Habsah Abdul Kadir, Kok Gan Chan, Bey Hing Goh

**Affiliations:** ^1^Jeffrey Cheah School of Medicine and Health Sciences, Monash University Malaysia, 46150 Bandar Sunway, Selangor Darul Ehsan, Malaysia; ^2^Division of Genetic and Molecular Biology, Faculty of Science, Institute of Biological Sciences, University of Malaya, 50603 Kuala Lumpur, Malaysia; ^3^Biomolecular Research Group, Biochemistry Program, Institute of Biological Sciences, Faculty of Science, University of Malaya, 50603 Kuala Lumpur, Malaysia

## Abstract

Ylang-ylang (*Cananga odorata* Hook. F. & Thomson) is one of the plants that are exploited at a large scale for its essential oil which is an important raw material for the fragrance industry. The essential oils extracted via steam distillation from the plant have been used mainly in cosmetic industry but also in food industry. Traditionally, *C. odorata* is used to treat malaria, stomach ailments, asthma, gout, and rheumatism. The essential oils or ylang-ylang oil is used in aromatherapy and is believed to be effective in treating depression, high blood pressure, and anxiety. Many phytochemical studies have identified the constituents present in the essential oils of *C. odorata*. A wide range of chemical compounds including monoterpene, sesquiterpenes, and phenylpropanoids have been isolated from this plant. Recent studies have shown a wide variety of bioactivities exhibited by the essential oils and the extracts of *C. odorata* including antimicrobial, antibiofilm, anti-inflammatory, antivector, insect-repellent, antidiabetic, antifertility and antimelanogenesis activities. Thus, the present review summarizes the information concerning the traditional uses, phytochemistry, and biological activities of *C. odorata*. This review is aimed at demonstrating that *C. odorata* not only is an important raw material for perfume industry but also considered as a prospective useful plant to agriculture and medicine.

## 1. Introduction

*Cananga odorata* Hook. F. & Thomson, which is commonly called ylang-ylang, is a fast growing tree and can found natively in tropical Asia such as Philippines, Malaysia, Indonesia, and some other islands of Indian Ocean, mainly the Comoro, Nossi Be, and Madagascar islands. This plant has been well-known for its fragrant flower and has been introduced to China, India, Africa, and America. Ylang-ylang essential oils have already been widely utilized in the food industry as well as in the perfume industry and aromatherapy. Primarily, the ylang-ylang essential oil is derived from the flower of the* C. odorata* plant via water or water and steam distillation. Ylang-ylang oil has been described to possess medium to strong initial aroma with fresh, floral, slightly fruity fragrant yet delicate. Furthermore, the flower is also described to produce intensely sweet scent which is similar to jasmine [1]. Ylang-ylang oil has been approved to be generally recognized as safe by Flavor and Extract Manufacturers Association (FEMA) and is widely used as flavouring agent and adjuvant. Currently, ylang-ylang oil can be found in various cosmetic and households products such as the massage oils, moisturizing creams, perfumes, and even scented candles. It is also believed that the medicinal properties exhibited by ylang-ylang oil are one of the main factors that contribute to its increasing popularity in the field of aromatherapy.

Although the uses of ylang-ylang oil and its safety as food ingredient have been also reviewed previously [2], during that time period the studies on the pharmacological activities of the* Cananga odorata *plant were still very limited. Basically, a very brief review was done covering the antibacterial, antifungal, amebicidal, and cytotoxic activities of the ylang-ylang essential oil [2]. Perhaps, it is due to the improvement in different biological assays and accessibility of chemical purification and identification techniques, and it has seemed greatly impacted by the research activities carried out by researchers. In particular, an apparent increase in the differential biological activities investigation on medicinal plants has enabled diversified applications of existing known natural products. For instance, the recent extensive explorations of differential pharmacological properties of ylang-ylang and their active compounds have significantly opened up its new commercial avenues for agriculture [3]. Insightfully there is a great increase in number of pharmacological studies done on* C. odorata *in recent years, particularly surrounding its biological properties and chemical components [4–8]. Therefore, the current review aimed to compile or summarize these important findings and further highlight the importance of* C. odorata* as a potential promising drug discovery candidate for future.

## 2. Taxonomic Classification and Nomenclature of* Cananga odorata*

Taxonomic classification and nomenclature of* Cananga odorata* are as follows:

 kingdom: Plantae, plants,
 subkingdom: Tracheobionta, vascular plants,
 superdivision: Spermatophyta, seed plants,
 division: Magnoliophyta, flowering plants,
 class: Magnoliopsida, dicotyledons,
 subclass: Magnoliidae,
 order: Magnoliales,
 family: Annonaceae, custard-apple family,
 genus:* Cananga* (DC.) Hook. f. & Thomson, ylang-ylang,
 species:* Cananga odorata *(Lam.) Hook. f. & Thomson, ylang-ylang.

## 3. Botany

### 3.1. Botanical Name

#### 3.1.1. Common Names

*C. odorata *is commonly known as ylang-ylang. The English names of* C. odorata* are ylang-ylang, perfume tree,* Cananga*, and cadmia. Meanwhile, the other common names for* C. odorata *are listed in Table 1.

### 3.2. Synonyms

According to the plant list, there are more than twenty synonyms which have been recorded for* C. odorata. *For instance,* Cananga mitrastigma *(F. Muell.) Domin,* Canangium mitrastigma *(F. Muell.) Domin,* Cananga odorata *var.* odorata*,* Cananga odoratum *(Lam.) Baill. ex King,* Canangium odoratum *(Lam.) Baill. ex King,* Canangium odoratum *var. velutinum Koord. & Valeton,* Cananga scortechinii *King,* Canangium scortechinii *King,* Fitzgeraldia mitrastigma *F. Muell.,* Unona cananga *Spreng.,* Unona leptopetala *DC.,* Unona odorata *(Lam.) Dunal,* Unona odorata *(Lam.) Baill.,* Unona odoratissima *Blanco,* Unona ossea *Blanco,* Uvaria axillaris *Roxb.,* Uvaria cananga Banks*,* Uvaria odorata *Lam.,* Uvaria ossea *(Blanco) Blanco, and* Uvaria trifoliata *Gaertn. [50].

## 4. Botanical Description and Distribution

*C. odorata *belong to the Annonaceae family, with 125 genera and 2050 species. To date, the* Cananga* genus consists of two species of plant, namely,* C. odorata* and* C. latifolia*.* C. odorata *is a perennial tropical tree which grows natively in South-East Asia countries such as Philippines and Malaysia, and it also occurs naturally in several Pacific islands including Australia. After that, it has been introduced into China, India, Africa, and America due to its economic importance [51].

**Figure 1:**
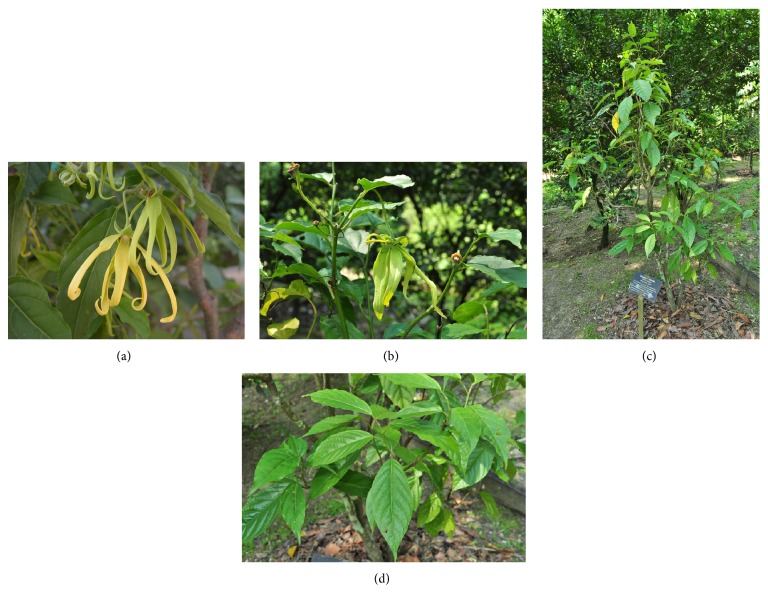
Morphology of* C. odorata*. (a) Mature* C. odorata *flower with yellow petals, (b) young yellowish-green* C. odorata *flower, (c) young* C. odorata* plant in Rimba Ilmu Botanic Garden, University of Malaya, and (d) leaves of* C. odorata *plant (images are obtained from Dr. Sugumaran (a) and Mr. Cheah ((b)–(d)) from University of Malaya).

The morphological features of the* C. odorata* plant are briefly described in Table 2 and illustrated in Figure 1. Basically,* C. odorata* is a medium-sized evergreen tree whichgenerally grows up to 15 meters height with long drooping branches [9].

## 5. Ethnomedicinal Uses

*C. odorata* has a variety of medicinal properties and traditional uses. The strongly fragrant yellow flower of* C. odorata* has been reported to be used to enhance the scent of coconut oil before being used for massage by Polynesians live in South Pacific islands [52]. In Java, the dried flowers of* C. odorata* are used to treat malaria and malaria-like symptoms. Similarly, it is also recognized as medicinal plants used against malaria traditionally in Vietnam [27]. Meanwhile, it has been also reported that the pounded fresh flowers paste being used to treat asthma. The flowers and bark of* C. odorata* are used to treat pneumonia and stomach ache by the local communities and traditional healers from Northern Mariana Islands [53]. In Indonesia, ylang-ylang oil is used to enhance euphoria feel during sex and also reduce sexual anxiety [54]. In line with the above mentioned traditional usage, ylang-ylang has been reported to be used as antidepressant to treat depression and nervousness. It has been also reported to have blood pressure lowering effect suggesting its potential use in managing hypertension [51].

According to both of the folks from India and islanders of the Indian Ocean, the leaves of* C. odorata *is believed to relieve itchiness by direct topical application and also to treat dandruff [55]. Indian has also used ylang-ylang oil to treat headaches, eye inflammation, and gout [52]. Apart from that, the traditional healers from Papuan New Guinea believe that by consuming the decoction of the heated inner bark of* C. odorata* is ableto treat gout [56]. Besides that, the bark of the plant is believed to be effective in treating stomach ailments. It is also being used as laxative by communities in Tonga and Samoa. Meanwhile, the Indian used the decoction of the bark of the plant to treat rheumatism, phlegm, ophthalmia, ulcers, and fevers [57].

## 6. Phytochemistry

The phytochemistry of* C. odorata *is well documented.* C. odorata *is well known for its essential oil. Essential oils are referred as the natural, complex, and volatile compounds which exhibit distinctive scent that are produced by aromatic plants as secondary metabolites [58]. Generally, the essential oils can be extracted from the aromatic plants by steam or hydrodistillation. However, various combinations of extraction methods are necessary to extract all the volatile phytochemicals present in the* C. odorata.* Besides the steam and hydrodistillation extraction methods, simultaneous steam distillation-solvent extraction and supercritical fluid extraction (SFE) were also developed to completely isolate most of the volatile secondary metabolites of ylang-ylang flower [13]. More advanced methods have been employed to analyze the volatile components of* C. odorata* due to several disadvantages presented by using distillation method such as time consuming and thermal degradation. For instance, Headspace-Solid Microextraction method coupled with Gas Chromatography-Mass Spectrometry (HS-SPME-GC-MS) was used to characterize all the volatile compounds of* C. odorata* flower at different stages of development [59].

**Figure 2:**
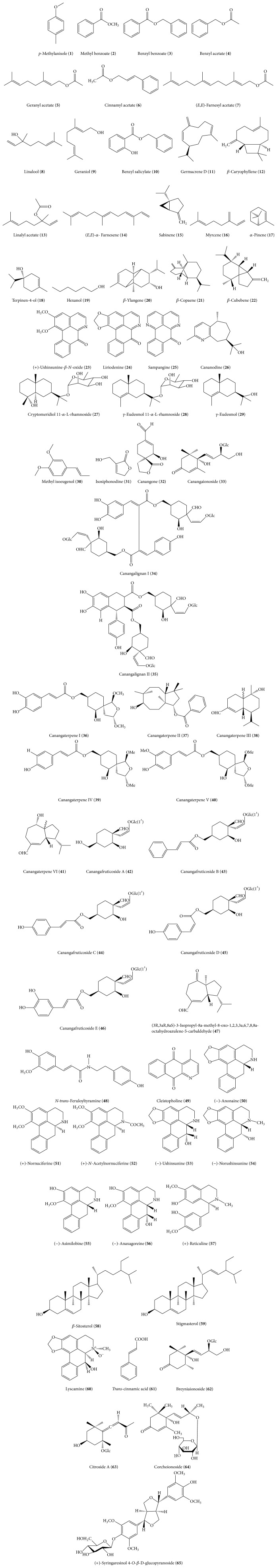
The molecular structures of the constituents isolated from different part of* C. odorata*.

Numerous chemical composition studies have been conducted on the essential oil of different parts of* C. odorata. *In one of the earliest reports, ylang-ylang essential oil was shown to contain monoterpene hydrocarbons, oxygen-containing monoterpenes, sesquiterpene hydrocarbons, oxygen-containing sesquiterpenes, benzenoids, acetates, benzoates, and phenols. To date, many compounds have been identified from the essential oil of ylang-ylang. Essentially, most of the compounds identified from the essential oil from different part of* C. odorata* plant are listed in Table 3. In 1986, a total of 52 compounds from the volatile, oxygenated, and hydrocarbon fractions of first grade ylang-ylang essential oil from Madagascar were identified by combined gas chromatography-mass spectrometry (GC-MS) and proton nuclear magnetic resonance (^1^H NMR). The study revealed that the main components identified from the oxygenated fraction of ylang-ylang essential oil were* p-*methylanisole** (1)**, methyl benzoate** (2)** and benzyl benzoate** (3)**, benzyl acetate** (4)**, geranyl acetate** (5)**, cinnamyl acetate** (6)** and (*E,E*)-farnesyl acetate** (7)**, linalool** (8)**, geraniol** (9),** and benzyl salicylate** (10) **and their molecular structures are shown in (Figure 2). Linalool** (8)** was shown to be main component present in oxygenated fraction (28%) that is responsible for the floral smell of ylang-ylang. Meanwhile, the hydrocarbon fraction of ylang-ylang oil consisted of mainly sesquiterpenes and monoterpenes whereby both germacrene D** (11)** and *β*-caryophyllene** (12) **represented 63% of the total hydrocarbon fraction of ylang-ylang oil [60]. *γ*-Muurolene** (13)** and (*E,E*)-farnesyl acetate** (7)** were both sesquiterpenes identified for the first time in ylang-ylang oil in [60]. In 2012, Benini and colleagues [15] demonstrated a total of 32 compounds which were not previously reported in ylang-ylang oil were detected from the* C. odorata *flower samples obtained from Grande Comore, Mayotte, Nossi Be, and Ambanja (Table 3). Furthermore, the characterization of ylang-ylang essential oils was further improved by the use of comprehensive two-dimensional GC coupled to time-of-flight MS (GC×GC-TOFMS) by the similar group of researchers. Brokl and colleagues [16] demonstrated that GC×GC-TOFMS was able to reveal more chemical components present in ylang-ylang flower (Table 3), suggesting that this technology is capable of providing better insight on chemical polymorphism as well as of studying the different parameters of “terroir effect” on phytoconstituents.

There are various factors that can influence the chemical composition and quality of the volatile secondary metabolites being extracted from the aromatic plants and flowers, particularly the extraction method, extraction time, and the flower conditions [13]. The essential oil extracted from the flower of* C. odorata* is the important main raw material for perfume industry. To date, four grades of ylang-ylang oil have been developed and are commercially available: the Extra, First, Second, and Third which contain different chemical compositions that determine the quality and uses of the oil. The Extra quality of ylang-ylang oil is highly recommended to be used in production of high-grade perfumes. This is because the Extra grade oil is rich in strongly odoriferous molecules such as linalool** (8)**,*p*-cresyl methyl ether (*p-*methylanisole)** (1)**, methyl benzoate** (2)**, benzyl acetate** (4),** and geranyl acetate** (5)** which are the volatile compounds that give the fragrance. Meanwhile the other grades contain increasing amount of sesquiterpene hydrocarbons which are less volatile such as *β*-caryophyllene** (12)**, germacrene D** (11),** and (*E,E*)-*α*-farnesene** (14)**. For instance, the First and Second grades are used in cosmetics. Lastly, the Third grade oil is being used for scenting of soaps. Besides depending on the fractionation based on distillation times, the chemical composition of ylang-ylang essential oils can be varied significantly depending on the stages of flower maturity [59, 61] and also geographical area which presents different environmental and agronomic conditions [15, 17]. Qin and colleagues [59] revealed high level of volatile polymorphism occurring along the 7 different flower development stages with only 52.45% of Bray-Curtis similarity value among all stages. The study showed that large amounts of volatile compounds including hydrocarbon, esters, and alcohols were detected in the full bloom stage of* C. odorata *which was the most suitable period for harvesting as those volatile compounds may have contributed to the aroma profile of* C. odorata *[59].

In term of geographical locations, by comparing the essential oil in the flower and fruits, the fruits of* C. odorata *from Cameroon were found to contain more abundant of monoterpenic essential oil such as sabinene** (15)**, myrcene** (16)**, *α*-pinene** (17),** and terpinen-4-ol** (18)** while the composition of essential oils present in the leaves of* C. odorata *from Cameroon was quite similar as compared to flower essential oil [10]. Similarly, another study [11] revealed that the composition of essential oil present in the leaves of* C. odorata *from Australia was relatively similar to the findings from Cameroon [10] but with larger amounts of hexanol** (19)** and absence of sabinene** (15)**. More recently, a study focused on the variation in the chemical profiles of essential oils from* C. odorata *among the Western Indian Ocean islands such as Union of Comoros, Madagascar, and Mayotte as they are known to be the current main producers of ylang-ylang essential oils [15]. The study revealed that there is a significantly high variation in terms of the proportion of essential oils constituents from each area of origin throughout the Western Indian Ocean islands [15].

With the advancement of bioinformatics, a number of genes responsible for volatile compounds biosynthesis pathway were elucidated with use of high-throughput RNA sequencing technology. Jin and colleagues [61] successfully characterized the functionality of four full-length of terpene synthases (TPSs), CoTPS1, CoTPS2, CoTPS3, and CoTPS4, extracted from yellow flower of* C. odorata*. One of the TPSs specifically known as CoTPS2 was found to be novel and multifunctional in which it could catalyze the synthesis of sesquiterpenes including *β*-ylangene** (20)**, *β*-copaene** (21)**, and *β*-cubebene** (22)** [61].

Besides the extensive studies on the phytoconstituents in the essential oil of* C. odorata*, the medicinal properties of nonvolatile constituents from the plant part have been investigated and reported as well. Several new compounds were isolated from the methanolic extract of the seeds of* C. odorata *in 1999 [62]. The study revealed a new stereoisomer of ushinsunine-*β*-*N*-oxide** (23)** and another 10 newly discovered compounds from this species for the first time. The isolated compounds from this extract are listed in (Table 4). For instance, liriodenine** (24)**, a cytotoxic oxoaporphine alkaloid, isolated from* C, odorata *was demonstrated to be a potent inhibitor of topoisomerase II in both* in vitro *and* in vivo *[62]. Besides the cytotoxic and antineoplastic activity of this compound, liriodenine** (24) **was also shown to be active against Gram-positive bacteria, yeast, and filamentous fungi. Sampangine** (25) **was another alkaloid isolated from the chloroform extract of the stem bark of* C. odorata *[19]. Literatures revealed that sampangine** (25)**, a copyrine alkaloid, exhibited antifungal, antimycobacterial, antimalarial activities and demonstrated cytotoxic effects toward human malignant melanoma cells [63]. A more recent study isolated and characterized four compounds from the fruits of* C. odorata *including cananodine** (26)**, new guaipyridine sesquiterpenes, cryptomeridiol 11-*α*-L-rhamnoside** (27)** and *γ*-eudesmol 11-*α*-L-rhamnoside** (28)**, both being new eudesmane sesquiterpenes, and lastly the *γ*-eudesmol** (29)**, a previously known eudesmane sesquiterpene [20]. The study also demonstrated that all the identified compounds displayed cytotoxicity against both hepatocarcinoma cancer cell lines, Hep G2, and Hep 2,2,15. Cryptomeridiol 11-*α*-L-rhamnoside** (27)** and *γ*-eudesmol** (29)** exhibited the most potent cytotoxic activity against Hep G2 and Hep 2,2,15 cell lines. Moreover, Ragasa and colleagues [33] revealed the isolation of methyl isoeugenol** (30)**, benzyl benzoate** (3),** and farnesyl acetate** (7)** from dichloromethane extract of air dried flower of* C. odorata*. The study further showed that the compound methyl isoeugenol** (30)** exhibited antibacterial and antifungal activities [33].

Furthermore, two lactone compounds have been isolated from the leaves and stems of* C. odorata *in conjunction with the searching for bioactive constituents from the* C. odorata *plant by a group of researchers [21]. Isosiphonodin** (31)** and a new spirolactone, named as canangone** (32)** were the two lactones isolated and identified from the acetone extract of dried leaves and stems of* C. odorata *[21]. Recently, a new megastigmane glucoside named as canangaionoside** (33) **was identified from the methanolic extract of the dried leaves of* C. odorata* [22]. Three new lignan dicarboxylates and six new terpenoid derivatives were also isolated by Matsumoto and colleagues from the methanolic extract of* C. odorata *flower buds [8, 23]. The new lignans isolated from the flower buds of* C. odorata* were named as canangalignans I** (34)** and II** (35)** [8], whereas canangaterpenes I, II, III, IV, V, and VI** (36–41)** were the six new terpenoid derivatives identified from the methanolic extract of* C. odorata *flower buds [8, 23]. They also indicated that one of the newly discovered terpenoids, canangaterpene I** (36),** exhibited potent antimelanogenesis activity [8]. Lastly, five usual monoterpene glucosides were also isolated and named as canangafruticosides A-E** (42–46)** by Nagashima and colleagues [24]. The chemical structures of both nonvolatile and volatile chemical compounds mentioned above are illustrated in Figure 2.

## 7. Bioactivities of* C. odorata*

Various biological activities of* C. odorata *have been extensively studied over the past decades. The detailed information of respective biological activities of* C. odorata *is being discussed as below. A summarized form of biological activities of* C. odorata *is then provided in Table 6.

## 8. Antimicrobial Activity

In the last decade, the emergence of multidrug resistance pathogens and strains with reduced susceptibility due to indiscriminate use of antibiotics has become a global concern [64] as the clinical efficacy of many existing antibiotics has been compromised. As a consequence, the therapy of the infections inflicted by the multidrug resistant pathogen is complicated and has led to substantial increased hospitalizations and greater risk for morbidity and mortality [65]. This issue has necessitated the scientist to screen for novel antimicrobial substances from various medicinal plant sources including the essential oils or the extracts from aromatic plants which have been reported to possess phytochemicals with antimicrobial activities [66]. The antimicrobial properties of the essential oils and extracts of* C. odorata* have been tested against various Gram-positive and Gram-negative pathogens as well as pathogenic fungi (Table 5).

Recently, the stem bark extracts of* C. odorata *obtained from Indonesia were shown to exhibit potent antimicrobial activities using the agar well disc diffusion assay. The study has demonstrated that* n-*hexane, ethyl acetate, and ethanolic extracts of* C. odorata* stem bark possessed good activity against* Propionibacterium acnes* and* Candida albicans*. The ethanolic extract of* C. odorata* at the dose of 400 *μ*g/well exhibited an inhibition zone of 19 ± 1.58 mm when tested against* P. acnes. *In fact, the activity index stands at 0.63 when being relative to the standard drug which is known as chloramphenicol [26]. Among the three extracts of* C. odorata *tested, the* n-*hexane extracts of* C. odorata *stem bark showed the highest inhibitory effect on* C. albicans* growth (17 ± 1.58 mm) at the dose of 100 *μ*g/well. It represents the activity index of 0.56 when being relative to the standard drug known as nystatin [26]. Meanwhile, in another study the researchers have taken the research to another next level of assessment. The purified constituents from the bark of plant were used to evaluate the antimicrobial activity on different species of bacteria. Besides, they have also examined the antifungi activities of these purified compounds [28]. In that particular research, those three tested compounds which are known as* O-*methylmoschatoline, liriodenine** (24),** and 3,4-dihydroxybenzoic acid were showing a significant antibacterial and antifungal activities at their respective dose at 200 *μ*g/disc and 400 *μ*g/disc. Among the three purified compounds, liriodenine** (24)** emerged as the strongest compound in exerting its antibacterial and antifungal activities against* Klebsiella *sp. and* C. albicans*, respectively [25].

On the other hand, a study was conducted specifically on the antimicrobial activity of the* C. odorata *leaf extracts. Three different extracts of* C. odorata *leaf were prepared and tested against selected Gram-positive and Gram-negative bacteria as well as different fungal strains [26]. The methanolic extract of* C. odorata *exhibited the highest antimicrobial activities as compared to petroleum ether and chloroform extracts. Moreover, the study also suggested that the Gram-negative bacteria demonstrated higher resistance than the Gram-positive bacteria against all the extracts of* C. odorata *leaf  [31]. Similarly, the antimicrobial activity of the essential oil of* C. odorata *showed high inhibitory effect with MIC_90%_ values at 0.23 mg/mL against* S. aureus *ATCC 25923 and clinical strains* S. aureus *[32]. However, both* E. coli *ATCC 25922 and* Pseudomonas aeruginosa* ATCC 27853 showed high resistance towards the essential oils of* C. odorata *which did not show inhibitory capacity up to the maximum concentration (27.12 mg/mL) tested in the study [32]. The study also has characterized the essential oil of* C. odorata* using GC-MS and revealed that the essential oil of* C. odorata *contained* trans-β*-caryophyllene (12.92%), linalool** (8)** (11.38%), germacrene D** (11)** (11.21%), benzyl acetate** (4)** (10.34%), and geranyl acetate** (5)** (9.87%) [32].

Meanwhile, the essential oil of* C. odorata *obtained from steam distillation was shown to exhibit weak antibacterial activity against* P. acnes *strains [67]. The inhibition zones of* C. odorata *essential oils against 5 strains of* P. acnes *were only ranging from 8.8 ± 0.7 mm to 9.5 ± 0.7 mm [67] which were relatively smaller as compared to ethanolic extract of* C. odorata *described in [26]. In effort of discovering the potential usage of ylang-ylang oil as alternative treatment for irritable bowel syndrome, three different antibacterial assays, namely, disc diffusion assay, turbidometric assay, and zone of clearance assay were conducted against* E. coli* [68]. However, essential oil of* C. odorata* demonstrated relatively low antibacterial activity against* E. coli* where the essential oil of* C. odorata *did not inhibit the growth of* E. coli *in either the agar plate or liquid culture and also did not show any killing ability against* E. coli* from the zone clearance assay [68]. The essential oil of* C. odorata *has also showed to exhibit no inhibitory effect against* Malassezia furfur*, which is a fungal pathogen associated with seborrheic dermatitis [69]. In contrast, another study demonstrated that the essential oils of* C. odorata *which contained germacrene D** (11)** (20%) and *β*-caryophyllene** (12)** (17%) exhibited slight fungicidal activity (12 ± 2 mm) against* Trichophyton mentagrophytes* TIMM2789 using agar diffusion assay [70].

The synergistic effects of ylang-ylang oil with different combinations of essential oils for treatment of microbial infections have also been reported. For an example, a study has proven that the combinations of ylang-ylang oil and thyme oil were significantly more effective against* S. aureus *ATCC 25923 and its synergistic effect was observed between both of the essential oils in which the inhibition zone was increased by 38.4% as compared to thyme oil alone [71]. However, a slight antagonism effect was then observed when ylang-ylang oil was used together with thyme oil against* Escherichia coli* ATCC 25922, the inhibition zone was reduced by 48.9% when compared to thyme oil alone [71]. Similarly, another study revealed that blended essential oil preparation which is comprised of lavender, clary sage, and ylang-ylang oils in the ratio 3 : 4 : 3 displayed a strong antibacterial and antifungal activities against* Staphylococcus aureus *ATCC 6538,* Staphylococcus epidermidis*,* Escherichia coli *ATCC 25923,* Pseudomonas aeruginosa *ATCC 9027, and* Candida albicans *ATCC 10231 [72]. The results also revealed that the preparation showed a synergistic antimicrobial effect against all the tested microorganisms. The increased antimicrobial activities displayed from the blended essential oil preparation as compared to the single or pure essential oil were believed to be contributed by the increased active components such as linalool** (8)** and linalyl acetate** (13)** present in the blended preparation [72].

Besides that, antiplasmodial activity of* C. odorata *was also evaluated by a group of researchers from Vietnam [27]. Nyugen-Pouplin and colleague revealed that the cyclohexane extract of* C. odorata *leaves at 10 *μ*g/mL exerted moderate antiplasmodial activity (75% inhibition) against* Plasmodium falciparum* FcB1 strain with IC_50_ value of 12.5 ± 3.9 *μ*g/mL [27]. The result of present study somehow ascertains the folkloric claim on* C. odorata *used as medicinal plant to treat malaria and malaria-like symptoms in Indonesia and Vietnam.

Overall, ylang-ylang oil and different extracts of* C. odorata* showed better antibacterial activities against Gram-positive bacteria than Gram-negative bacteria. For instance,* S. aureus *showed high susceptibility to the essential oils and extracts of* C. odorata *as compared to other tested Gram-negative bacteria. Studies also showed that* C. odorata *exhibited a remarkable antifungal activity. Disc and well diffusion assay were the most common tests being employed to evaluate the antimicrobial activity of the essential oil and extracts of* C. odorata*. Although the antimicrobial activity of* C. odorata *tested was not as potent as other essential oils and extracts of other plants, studies have demonstrated that the synergistic effects observed from the combinations of different medicinal plants and herbs may potentiate the antimicrobial activities against pathogens.

## 9. Antibiofilm Properties

Many bacteria possess the ability to form biofilm, which is a slimy layer comprised of bacterial cells that protected by self-synthesized matrices of polysaccharides and proteins that allows attachment to various surfaces such as polystyrene, glass, and stainless steel in different environments [73]. The formation of microbial biofilms poses a significant challenge to current clinical and industrial settings as microbial biofilms are associated with dramatically enhanced tolerance towards most antimicrobial agents and disinfectant chemicals as well as the body's immune system. Hence, the increased resistance developed by the formation of biofilm contributes to the chronicity of microbial infections and leading to therapy failure [74]. Although many approaches have been implemented in controlling biofilms, the discovery for novel, natural, and effective antibiofilm agents is still undergoing in order to cope with the increased biofilm-associated health problems. The plant-derived essential oils have been explored extensively to combat biofilm formation. For instance, oregano oil [75], eucalyptus oil [76], tea tree oil [77], cinnamon oil [73], and lemon grass [78] have been demonstrated to exhibited potent antibiofilm activities against wide range of bacteria. Recently, the antibiofilm activity of cananga oil also has been evaluated in several studies [5]. A study revealed that ylang-ylang oil exhibited strong antibiofilm activity at dose-dependent manner against biofilm formation of* Staphylococcus aureus *ATCC 6538 [5]. The study utilized a static biofilm formation assay, confocal laser microscopy, and also scanning electron microscopy to examine the effect of cananga oil on biofilm formation of* S. aureus *[5]. It was found that 0.01% (v/v) of ylang-ylang oil showed more than 80% inhibition against biofilm formation of* S. aureus* as compared to the control group but did not inhibit the growth of* S. aureus*. Furthermore, the study also suggested that both* cis-*nerolidol and* trans-*nerolidol were the constituents in ylang-ylang oil that is responsible for the inhibition of biofilm formation [5]. Furthermore, another study combined the unique properties of magnetic nanoparticles which have been reported to be effective delivery systems with the ylang-ylang oil as a coating agent for surfaces of implantations with the intention to reduce the development of biofilm [79]. The study has shown the incorporation of ylang-ylang oil with iron oxide@C_14_ nanoparticles effective in inhibiting the initial adherence phase of clinical strains of both* S. aureus *and* Klebsiella pneumonia* with more than 2-log reduction to the coated catheter specimens [79]. The results of the study have suggested the potential use of ylang-ylang oil in nanobiosystems with antibiofilm activity [79].

## 10. Antioxidant Properties

The generation of free-radical intermediates through oxidative stress has been known to cause disturbances in metabolic processes. They are known to be responsible for cellular injuries and disease formation due to the destruction of unsaturated lipids, proteins, and DNA. The implications of oxidative damage have been linked to many human diseases such as cancer, cardiovascular diseases, inflammatory processes, cataracts, and even the normal ageing process [80]. Recently, natural occurring antioxidants have been of great interest because of people's concerns over the use of synthetic antioxidants such as butylated hydroxyanisole (BHA), butylated hydroxytoluene (BHT), propyl gallate, and tert-butylhydroquinone (TBHQ) which may have adverse effects on human health [81]. The antioxidant activity of* C. odorata *extracts was evaluated using DPPH assay to determine the free radical scavenging abilities of the extracts. The result of the study revealed that the ethyl acetate extract of the stem bark of* C. odorata *exhibited the highest percentage of DPPH inhibition (79%) as compared to other tested plant extracts [26]. Besides the DPPH assay, the antioxidant activity of methanolic extract of* C. odorata *leaves was also determined by ferric ion reducing power assay. The extract showed a total of 290.0 ± 13.1% of ferric reducing power at 0.5 *μ*g/mL [34].

Normally, a series of antioxidant assays will be utilized to examine different aspects of antioxidant property of plant extract. In a particular study, antioxidant activity of the* C. odorata *essential oils was assessed using free radical-scavenging, *β*-carotene bleaching, and the luminol-photochemiluminescence assays [30]. The results of the study revealed that all the tests indicated that essential oil of* C. odorata* was a decent source of antioxidant. In detail, the free radical-scavenging activity of* C. odorata *was 63.8 ± 0.45% of DPPH inhibition and the value was twice higher than that of trolox, one of the reference oils with potent antioxidant activity. Furthermore, the results were further supported by the lipid peroxidation inhibitory activity displayed by the essential oil of* C. odorata* (75.5 ± 0.51% inhibition) in the *β*-carotene bleaching test. The luminol-photochemiluminescence assay also showed that the essential oil of* C. odorata *exhibited effective superoxide radical scavenging activity [30]. Consistently, the essential oils extracted from the flower of* C. odorata* originated from Madagascar also exhibited good DPPH radical scavenging activity (80.06 ± 0.02%) [82].

## 11. Antivector/Insecticidal/Antipest Properties

Dengue disease, which is a tropical and subtropical mosquito-borne viral illness, has become a public health concern worldwide. According to World Health Organization [83], statistics showed that approximately 2.5 billion people live in countries that are endemic for dengue and estimated that 50–100 million infections occur annually. There was dramatic increase in the number of reported cases of dengue disease in Malaysia, particularly in 2013 where incidences of dengue fever (143.27 per 100,000 population) were doubled as compared to 2012 (72.2 per 100,000 population) [84, 85]. However, the prevention of dengue fever is only restricted to managing the vector* Aedes aegypti* due to absence of effective prophylactics or vaccine against the infection. Generally, synthetic insecticides such as DDT and other chlorinated hydrocarbons are used to control the mosquitoes. However, the continuous application of these synthetic compounds has resulted in the development of resistant strains of mosquito vectors particularly* A. aegypti*. Hence, considerable study has shifted the interest towards natural products which may be effective in controlling the vector population. Larvicidal, ovicidal and repellent properties of essential oils and extracts from several plant species against mosquito vector have been evaluated including* Cananga odorata*. Studies have demonstrated that the essential oil of* Cananga odorata* possessed repellent properties as well as oviposition-deterrent and ovicidal activities against several mosquito species. In 2011, the insecticidal activity of the essential oils of* Cananga odorata *prepared in soybean oil was evaluated using standard WHO susceptibility testing protocols. It was found that the essential oil extracted from ylang-ylang flower at doses of 1%, 5%, and 10% (w/v) exhibited low insecticidal activity and knockout rate against all three types of adult mosquito species including the* Aedes aegypti*,* Culex quinquefasciatus,* and* Anopheles dirus*, with LC_50_ values of 9.77%, 8.82%, and 4.99%, respectively [35]. Targeting the breeding sites of mosquitoes is one of the effective strategies to control and eradicate the population density of the mosquito vectors. Furthermore, the mosquito life cycle can be disrupted by preventing them from undergoing oviposition which is an important event shaping both individual fitness and vectorial capacity in life history of mosquito [86]. Study has revealed that the essential oil of* C. odorata* may serve as a potential mosquito egg control agent against the species of* Aedes aegypti, Anopheles dirus, *and* Culex quinquefasciatus*. It was found that 10%* C. odorata* in soybean oil exhibited significantly high oviposition-deterrent and ovicidal activities against all three tested mosquito species. However, further study was suggested by the author as most of the results obtained from previous studies related to oviposition-deterrent and ovicidal were not promising and most of the mosquito eggs were shown to be tolerant to the action of insecticides [36]. Besides that, larvicidal and pupicidal activities of the essential oil of* Cananga odorata *against three immature stages of* Aedes aegypti, Anopheles dirus, *and* Culex quinquefasciatus* were evaluated [87]. Although the essential oil of* C. odorata *was not as effective as the essential oil of* Syzygium aromaticum* which was the most effective against all immature stages of the three tested mosquito species in the study, higher larvicidal and pupicidal activities were demonstrated by* C. odorata *essential oils against all immature stages of both* C. quinquefasciatus *and* Anopheles dirus* as compared to* A. aegypti* [87]. Similar results were also demonstrated in [37] whereby the essential oils of* C. odorata *exhibited low larvicidal activity against* A. aegypti* with only 40.0 ± 4.1% mortality observed at dose of 0.1 mg/mL. In a more recent study on the larvicidal activity of* C. odorata,* the chemical composition of the essential oils was determined with GC-MS and was evaluated together with the insecticidal activity of the plants against the third and fourth instar stage of* A. aegypti *[38]. The study revealed that the essential oils of* C. odorata *demonstrated moderate insecticidal activity with LD_50_ at 52.96 ppm against the immature stage of* A. aegypti* among the plants evaluated [38]. Benzyl acetate** (4)**, linalool** (8),** and benzyl benzoate** (3)** were the three major compounds identified from the essential oils of* C. odorata *with the percentage of 18.2%, 14.1%, and 12.3%, respectively [38].

Moreover, recent study also showed that* C. odorata *oil prepared in ethyl alcohol possessed larvicidal effect and oviposition-deterrent activity as well against house fly,* Musca domestica*. The control of house fly is also essential as it is known to be a serious disease causing pest which can transmit pathogenic organisms such as protozoa cysts, parasites, enteropathogenic bacteria, and enterovirus to human and livestock. The study demonstrated that* C. odorata *oil exhibited larvicidal effect against the 3rd instar larvae of house fly with median lethal time (LT_50_) value of 52.08 hours and LC_50_ value of 29.36% as compared to cypermethrin (10% w/v), a common chemical insecticide, with LT_50_ and LC_50_ of 31.63 hours and 11.45%, respectively [39]. Furthermore, excellent oviposition-deterrent activity was also demonstrated by* C. odorata *oil with 100% effective repellency value against the female house fly from undergoing oviposition at both concentrations of 1.65 *μ*L/cm^2^ and 3.30 *μ*L/cm^2^ [39].

Seo and colleague [40] also assessed the insecticidal activities of the essential oil from* C. odorata *flower against Japanese termite,* Reticulitermes speratus* Kolbe. The fumigation bioassay employed by the study [40] found that the essential oil* C. odorata* at 2 mg/filter paper resulted in cumulative mortalities of 18.0 ± 5.8% and 94.0 ± 4.0% of the termites after 2 and 7 days of exposure, respectively.

Besides that, the essential oil of* C. odorata *leaves has been demonstrated to possess antipest properties as well and could be considered to have the potential to be developed as possible natural fumigant or insecticide for control of insect associated with storage products [41]. The study showed that topical application of essential oil of* C. odorata *leaves exhibited toxicity against* Sitophilus zeamais*, which is a pest associated with corn storage, with a LD_50_ value of 33.14 *μ*g/adult [41]. Furthermore, fumigant activity of* C. odorata *essential oil against* S. zeamais* was also evaluated using vapour phase toxicity bioassay. The results showed that the essential oil of* C. odorata *leaves exhibited fumigant toxicity against* S. zeamais *with a LD_50_ value of 14.77 mg/L. The study also suggested that linalool** (8)**, which is a competitive inhibitor of acetylcholinesterase, might be the active component that accounted for the insecticidal activity of* C. odorata *essential oil [41].

## 12. Insect-Repellent Properties

Insect repellent is known to be one of the most effective ways to reduce the transmission of vector-borne diseases especially from mosquito [88]. With the fact that no effective vaccine against dengue is available, protection from mosquito bites could be only achieved by preventing physical contact with mosquitoes using repellents. Studies have indicated that the essential oil of* Cananga odorata* prepared in soybean oil possessed certain degree of repellent activity against the adult mosquito of* A. aegypti, A. dirus, *and* C. quinquefasciatus* with the ED_50_ of 0.045, 2.149, and <0.003 mg/cm^2^. The essential oil of* Cananga odorata* also demonstrated a moderate time of protection against* A. aegypti*,* A. dirus,* and* C. quinquefasciatus* at a duration of 8.4, 24.0, and 60.0 minutes, respectively [42] even though the protection time of DEET-based repellent which remains the gold standard of protection, in which 23.8% DEET showed a complete 5 hours protection against* A. aegypyi* bites [89]. Meanwhile, another study revealed that the protection time was improved by* C. odorata *oil prepared in ethyl alcohol at 0.33 *μ*L/cm^2^ against* A. aegypti* and* C. quinquefasciatus* with 86.67 ± 10.40 and 126.0 ± 15.77 minutes, respectively [43]. Similarly, a more recent study revealed that essential oil of* C. odorata *prepared in coconut oil at 0.33 *μ*L/cm^2^ showed a better activity with 98.9% protection from bites of* A. aegypti *with an improved protection time for 88.7 ± 10.4 minutes among the three tested diluents [4]. The discrepancy between the studies may be due to many factors that might affect the efficacy of the repellent such as the species and density of mosquito, the age, gender, and biochemical attractiveness of the subject as well as the experimental conditions [43]. Most of the studies indicated above have shown that indeed the essential oils of* C. odorata* demonstrated good mosquito-repelling properties against different species of mosquitoes.

Besides the repellent activity against mosquito, the essential oil of* C. odorata *leaves has been shown to exhibit repellent activity against* Tribolium castaneum*, a red flour beetle which is known to be the pest associated with stored products, hence protecting the stored products from insect damage [3, 90]. The essential oil of* C. odorata *leaves was shown to have the strongest repellent effect against* T. castaneum* at concentration of 5 *μ*L per gram of oats as compared to other tested essential oils in the study [90]. Caballero-Gallardo and colleagues [3] also demonstrated that essential oil from* C. odorata *exhibited the highest percentage of repellency of 98% at 0.2 *μ*g/cm^2^ after both exposure times of 2 and 4 hours against* T. castaneum*, suggesting that it can be considered excellent candidates as natural repellents.

## 13. Antimelanogenesis

Melanin production or melanogenesis determines the skin color of animals and humans. Although melanogenesis is a major protective mechanism against UV-induced skin injury, the excessive production of melanin due to extensive UV exposure can lead to dermatological disorders. There has been increasing interest towards the findings of alternative herbal for treatment of hyperpigmentation because of the increased reports of potential mutagenicity and cases of ochronosis due the use of tyrosinase inhibitor such as hydroquinone, which is one of the most widely prescribed compounds found in nowadays cosmetic products and depigmenting agents [91]. Recently, the methanolic extract of the flower buds of* C. odorata* was found to exhibit inhibitory effect against melanogenesis [8]. The inhibitory effect of the constituents extracted from the flower buds of* C. odorata *was demonstrated by the detection of the melanin content in theophylline-stimulated B16 melanoma 4A5 cells via photometric method at 405 nm [8]. The study indicated that several compounds isolated from the methanolic extract of the flower buds of* C. odorata* displayed the inhibitory effect on melanogenesis and without induced any cytotoxicity to B16 melanoma 4A5 cells. Furthermore, there were two terpenoid derivatives (compound 5, canangaterpenes I** (36)** (IC_50_ = 3.6 *μ*M) and compound 12, (3R,3aR,8aS)-3-isopropyl-8a-methyl-8-oxo-1,2,3,3a,6,7,8,8a-octahydroazulene-5-carbaldehyde** (47)** (IC_50_ = 2.5 *μ*M)) exhibited stronger activity in inhibiting the production of melanin than the positive control, arbutin (IC_50_ = 174 *μ*M) [8]. Also, the study found that lignans with a catechol moiety and without the glucosyl moieties are essential for the inhibitory activity of melanogenesis [8]. Therefore, the study showed that the flower buds of* C. odorata* contain terpenoid derivatives which may have high potential for the treatment of skin disorder or cosmetic industry. Besides that,* N-trans*-feruloyltyramine** (48)**, which was a phenylpropanoid isolated from the methanolic extract of the seeds of* C. odorata* [18], may be another constituent that is responsible for the suppression of melanogenesis as this compound has been reported to show more potent inhibitory activity on the expression of tyrosinase protein (an important enzyme in melanin biosynthesis) in mouse B16 melanoma cells than the kojic acid (a tyrosinase inhibitor) [92]. In contrast, a study showed that the aqueous extract of* C. odorata *did not inhibit dopachrome formation (−19.8 ± 0.7%) which indicated that no antityrosinase activity exhibited by the aqueous extract of* C. odorata *[93]. These observations deduced that the inhibitory effects on melanogenesis of* C. odorata *extracts are involving the regulation of tyrosinase gene expression rather than the direct inhibition of tyrosinase activity.

## 14. Anti-Inflammatory Properties

Inflammatory diseases such as rheumatism, arthritis, and pelvic inflammatory disease continue to be one of the major health concerns worldwide. Traditional remedies have been known to be one of the most common ways to treat inflammatory diseases. For instance, the folkloric practice of treating joint pain with Willow (*Salix alba*) bark has led to the discovery of aspirin as the most commonly used pain reliever for 100 years [94]. Despite that, many steroidal and nonsteroidal anti-inflammatory drugs (NSAIDs) have been introduced to treat various inflammatory disorders. However, adverse side effects including renal problems, gastrointestinal irritation, and even myocardial infarction and strokes have been reported due to the prolonged use of steroidal and NSAIDs [94]. Hence, researchers have becoming more interested in evaluating the anti-inflammatory potential of plants traditionally used for relieving aches, asthma, and pains for the discovery and development of potent anti-inflammatory drugs. Traditionally, different parts of* C. odorata *plants have been exploited and used to treat fever, asthma, and pains. Several scientific evaluations were also conducted on the anti-inflammatory activities of* C. odorata.*

Wei and Shibamoto [6] demonstrated that the essential oil of* C. odorata* displayed anti-inflammatory properties using 15-lipoxygenase inhibitor screening assay. Lipoxygenases are enzymes that catalyze the metabolism of arachidonic acid in producing metabolites that regulate inflammatory response in mammals. The essential oil of* C. odorata* showed strong lipoxygenase inhibitory effect (~80%) at a concentration of 0.5 *μ*g/mL and also exhibited lipoxygenase inhibitory activity that appeared similar to nordihydroguaiaretic acid, a standard lipoxygenase inhibitory chemical, in a reverse dose-response manner [6]. The study also suggested that the lipoxygenase inhibitory effect was accounted by the major constituents present in the essential oils such as linalool** (8)**, linalyl acetate** (13),** and other volatile constituents [6]. These chemical constituents are normally found to possess anti-inflammatory activities in previous experimentations [95]. Furthermore, the methanolic extract of* C. odorata *leaves was shown to possess moderate inhibitory effect (97.9 ± 14.6%) on nitric oxide release in macrophage RAW264.7 cells with low cytotoxicity (cell viability: 89.7 ± 0.5%) at 50 *μ*g/mL [34]. Although nitric oxide is produced to act as a defense and regulatory molecule during inflammatory reactions, it may damage normal tissue when it is excessively produced [34, 96]. Overall, the findings indicated that the methanolic extract of* C. odorata* leaves may be a potential anti-inflammatory agent as the release of nitric oxide by macrophages has long been associated with inflammation.

Besides the* in vitro *studies mentioned above, the anti-inflammatory activity of* C. odorata *also had been evaluated in experimental animals recently. The ethanolic extract of* C. odorata *fruit was shown to exhibit significant anti-inflammatory activity in the carrageenan induced paw edema model of Wistar albino rats with LD_50_ > 2000 mg/kg [44]. The acute oral toxicity study indicated that the ethanolic extract* C. odorata *fruit was more effective in inhibition of paw volume (62.9%) at dose of 100 mg/kg than aspirin with inhibition of 60.14% at dose of 300 mg/kg. Furthermore, the author of the study suggested that anti-inflammatory effect of the extract might be due to the presence of flavonoids and tannins which is responsible for inhibiting both cyclooxygenase and lipoxygenase pathway [44].

## 15. Sedative, Relaxing, and Harmonizing Effects

The essential oil obtained from the leaves of* C. odorata *using hydrodistillation method extraction was shown to possess sedative effect and certain degree of physiological influence on human [45]. The study indicated that sniffing* C. odorata *oil decreased the systolic and diastolic blood pressure of human from 106.43 ± 11.51 mmHg to 105.20 ± 10.72 mmHg and 70.60 ± 10.53 mmHg to 69.20 ± 11.71 mmHg, respectively, demonstrating that the oil exhibited sedative effect. The results were further supported by the decreased pulse rate after sniffing* C. odorata* (73.40 ± 7.38 bpm) as compared to the control (75.33 ± 7.55 bpm). On top of that, the study also found that* C. odorata *essential oil exhibited relaxing effect on the volunteers after sniffing the oil, reducing the stress index from high level (73.33 KU/L) to medium level (49.50 KU/L). The stress level was also determined by measuring the alpha brain wave of the volunteers and the results showed that sniffing the essential oil of* C. odorata* increased an individual's alpha brain wave or also decreased one's stress level [45]. Similar results were also evidenced by Hongratanaworakit and Buchbauer [46] whereby the inhalation of ylang-ylang oil significantly decreased both systolic and diastolic blood pressure and pulse rate, indicating that inhalation of ylang-ylang oil decreased autonomic nervous system arousal. Besides that, the similar study [46] evaluated the effect of inhalation of ylang-ylang on the behavioural level of subjects in the aspect of alertness and attentiveness. The study demonstrated that the subjects felt more attentive and more alert after inhaling the oil, suggesting that the effect of inhalation of ylang-ylang oil is characterized as “harmonization” which resulted in uncoupling of physiological (reduced ANS arousal) and behavioural arousal process (increased behavioural activation) [46]. Meanwhile, the similar group of researchers found that transdermal administration of ylang-ylang oil to healthy subjects resulted in both decreased physiological arousal and deactivation of behavioural level whereby the subjects experienced more calm and relaxed after transdermal administration [97]. The findings of these studies indicated that the differential effects of essential oils depend on the route of administration whereby inhalation and percutaneous administration of the essential oils give different pharmacological and psychological effects either with or without involving the olfactory processing [97]. Moreover, the most recent study evaluated the sedative effects of ylang-ylang oil with the use of sphygmomanometer and electrocardiogram (EKG) to determine the blood pressure and heart rate, respectively, of the subjects after the inhalation of the fragrance of the oil [98]. Similarly, this study also indicated that ylang-ylang oil showed sedative effectiveness where declination of 12-lead EKG demonstrating decreased heart rate was observed in the group treated with ylang-ylang oil [98]. Overall, the available studies have shown that the essential oil of* C. odorata *indeed possess sedative, relaxing, and also harmonization effects on human and also explained its usefulness in aromatherapy and medicine such as reduction of blood pressure or relief of depression and stress in human.

## 16. Effects on Mood and Cognitive Performance

Studies have shown that the mood and cognitive performance of a healthy individual can be modulated by aromas of essential oils. A study revealed that ylang-ylang aroma acted significantly different on the cognitive performance of the healthy volunteers as compared to the control group and the peppermint aroma [47]. Ylang-ylang aroma produced a reduced alertness mood and increased calmness of the healthy volunteers but absence in the enhancement of cognitive performance and also lengthened processing speed [47]. Ishiguchi and colleagues evaluated the effect of inhalation of ylang-ylang essential oil by detecting the electroencephalography background activity of the volunteers [99]. They revealed that alpha 1 (8–9.9 Hz) brain waves which present in deep relaxation was increased significantly during inhalation of ylang-ylang essential oil and also reduced alertness mood of the volunteers [99]. Thus, Ishiguchi and colleagues suggested that the lowering effect of alertness and increased alpha 1 brain waves may be the physiological basis for relaxation effect of aromatherapy with ylang-ylang [99]. Furthermore, reduction of the amplitude of auditory P300 which is associated with the higher cognitive processing was observed in healthy volunteers during inhalation of ylang-ylang aroma, suggesting a relaxing effect of aroma on cognitive function. Watanabe and colleagues [100] elucidated the effect of ylang-ylang aroma on the auditory P300 of healthy individual and patient with temporal lobe epilepsy (TLE) who have impaired odor identification. The study demonstrated exposure to ylang-ylang aroma prolonged latencies of P300 in both control and TLE groups while only significant reduction of P300 amplitudes in healthy volunteers was observed. The absence of P300 amplitudes reduction in TLE patients suggested that their information processing was not altered during the exposure to ylang-ylang aroma or the fact that TLE patients had lower P300 amplitudes under odourless condition as compared to the controls [100].

## 17. Spermatotoxic Properties

Overpopulation is known to be a global issue and public health concern. The ever-increasing human population causes various detrimental effects including environmental degradation, poverty, and rise in unemployment. Therefore, many studies have been focusing on the discovery and development of novel and more potent contraceptive. Currently, medicinal plants have also received huge attention for its use as contraceptives due to their little side effects.* C. odorata *was also found to possess spermicidal activity in both* in vitro* and* in vivo *study [48]. In the* in vitro* study, the sperms obtained from healthy male rats were immobilized by 50% ethanolic extract of root bark of* C. odorata* within seconds. In the* in vivo *study, the administration of crude extract at 50 mg/100 g body weight/day reduced the motility of sperm of the rat significantly (5 ± 0.38 seconds) as compared to the control rat (30 ± 1.98 seconds). Furthermore, reduced sperm count and 94% abnormal sperm morphology were also observed when 100 mg/100 g body weight/day of crude extract was administrated into rats. The biochemical findings of the study indicated that the crude extract of* C. odorata *root bark reduced the production of testosterone, altered the metabolism of stored spermatozoa in the testes, and led to the deficiency in nutrients for proper sperm maturation [48]. Comparison between the antifertility effects of extract of* C. odorata *bark and gossypol which is a well-studied antifertility agent has been conducted as well [49]. The study suggested that* C. odorata *bark extract may be a better antifertility agent than gossypol as reversibility in the motility of sperm was observed in the* C. odorata *treated group after withdrawal of the extract. The study also managed to isolate the active component of the extract and was determined as a 52 kd protein which immobilized the sperm* in vitro *within seconds [49].

## 18. Antihyperglycemic Effects and Antidiabetic Complications Properties

Diabetes mellitus is a common metabolic disorder characterized by chronic hyperglycemia, as a result from defects in insulin production and insulin action. Currently, there is a need to develop safe and treatment for diabetes as most of the available medications have several adverse effects. According to [101], it was found that approximately 1200 species of plants were used as traditional medicine in treating diabetes globally. The leaves and stem extracts of* C. odorata* were found to exhibit alpha-amylase inhibitory effects [7]. Both leaves and stem extracts of* C. odorata *demonstrated 22.6 ± 1.3% and 25.3 ± 3.3% inhibition, respectively, at 7.8 *μ*g/mL on porcine pancreatic *α*-amylase enzyme. However, both leaves and stem extracts of* C. odorata *did not exhibit any inhibitory effect on *α*-glucosidase enzyme [7]. The results of the study suggested that the extracts of* C. odorata *may have the potential to be used as *α*-amylase inhibitor in managing postprandial hyperglycemia. Another study revealed that several terpenoid derivatives and flavonoids isolated from the flower buds of* C. odorata* possessed inhibitory effects on aldose reductase [23]. Aldose reductase is an enzyme in the polyol pathway that reduces glucose to sorbitol with the use of NADPH. The accumulation of intracellular sorbitol as a result of abnormal activation of polyol pathway may lead to chronic complications of diabetes such as diabetic neuropathy, retinopathy, nephropathy, and cataract [102]. Hence, the inhibition of aldose reductase activity may help in preventing diabetic complications. The study showed that canangaterpene I, (*E*)-[(1R,3R,5S,6S,8S)-6-hydroxy-1,3-dimethoxy-2- oxaspiro[4.5]decan-8-yl]methyl caffeate, and canangafruticoside E exhibited potent inhibitory effects on aldose reductase with IC_50_ at 1.2, 1.5, and 0.8 *μ*M, respectively, with comparison to a reference compound, chlorogenic acid with IC_50_ at 0.7 *μ*M [23].

## 19. Commercial Uses

Many patents exist which describe the commercial application of ylang-ylang oil. Of the 866 references to “ylang-ylang” that were located by “Scifinder,” 533 of these (61.5%) were patents. At the time of writing, recent patents involving ylang-ylang oil showed that a majority of the inventions have focused on field of health products and cosmetic uses. Ylang-ylang oil has been reported to be ingredients for many cosmetic products such as skin care products [103, 104], hair protecting products [105], hair growth promoter [106], and sunscreen compositions [107]. Besides that, the essential oils of* C. odorata *also present applications in agriculture and food industry. The essential oil of* C. odorata *has also been reported to be one of the ingredients for an invention used as repellent against insects, arachnids, and other arthropods [108]. In addition,* C. odorata* oil has also been incorporated as one of ingredients into a beverage formulations for the use as nutritional supplement [109]. All these patents demonstrate a strong commercial value and wide range of uses of* C. odorata *essential oil.

## 20. Conclusion

Extensive literature survey demonstrated that* C. odorata *is a medicinal and aromatic plant with a vast spectrum of pharmacological activities having considerable importance in agricultural and consumer products industries. The constituents such as* O-*methylmoschatoline, liriodenine** (24)**, 3,4-dihydroxybenzoic acid, germacrene D** (11),** and *β*-caryophyllene** (12)** have been recognized as the bioactive molecules that possess antimicrobial activities. Linalool** (8)** is another compound that has been shown to exhibit insecticidal and anti-inflammatory activities. Besides that, it has been experimentally proven that* C. odorata *also possess antibiofilm, antioxidant, antidiabetic, antifertility, antimelanogenesis, insect-repellent, antihyperglycemic, sedative, and relaxing properties. And overall, this review emphasizes the potential of* C. odorata* to be used as new therapeutic drugs and also provides sufficient baseline information for future works and commercial exploitation.

## Figures and Tables

**Table 1:** The common names of *C. odorata *from different regions.

Regions	Common names
General	Ylang-ylang, perfume tree, *Cananga*, and cadmia (English)

Oceania	Canang odorant (French) Chiráng, irang (Palau) Derangerang, derangirang (Nauru) Ilahnglahng, ilanlang (Kosrae) Ilang-ilang, alang-ilang (Guam) Ilangilang, lengileng, alangilang, pur-n-wai, pwurenwai, seir en wai (Pohnpei) Ilanilan (Marshall Islands) Lanalana (Hawai‘i) Makosoi, mokohoi, makasui, mokosoi (Fiji) Mohokoi (Tonga) Moso‘oi (Samoa) Moto‘i (French Polynesia) Moto‘oi, mata‘oi, mato‘oi (Cook Islands, Niue, Tahiti) Motoi (Marquesas-Nuku Hiva, Niue) Mutui (Marquesas-Fatu Hiva) Pwalang (Puluwat atoll) Pwanang, pwuur, pwalang (Chuuk) Sa‘o (Solomon islands: Kwara‘ae)

South East Asia	Ilang-ilang, alang-ilang (Philippines) Sagasein, kedatngan, kadatnyan (Myanmar) Kernanga (Indonesia) Fereng, kradang naga (Thailand) Kenanga, chenanga, ylang-ylang (Malaysia)

India	Apurvachampaka, chettu sampangi, karumugai (India)

Adapted from [9] with slight modifications.

**Table 2:** The morphological features of *C. odorata* leaves, stems, flowers, fruits, and seeds.

Part	Descriptions
Leaves	Colour: dark shiny green (above), duller and lighter green (beneath) Arrangement: alternate, single plane along twigs Length: 9–21 cm; width: 4–9 cm Shape: ovate-oblong to broadly elliptic with wavy margin; rounded and unequal base; acuminate apex

Twigs/petiole	Petiole colour: light green; twig colour: light green (young), brown (old) Petiole length: 6–15 mm

Flowers	Odor: highly fragrant Length: 7.5 cm Arrangement: hanging axillary in a group of 4–12 flowers with umbellate arrangement; scattering around the older parts of twigs Pedicels: short, 1–2.5 cm long Calyx: three, broad, pointed, and hairy Petals: six, slightly thicken, twisted, pointed, hairy, 4–6 cm long, green (young), yellow to yellowish-brown (mature)

Fruits	Colour: dark green to black (ripe) Shape: ovoid Length: 1.5–2.3 cm long

Seeds	Shape: hard, flattened, ovoid, and pitted Size: 6 mm diameter Colour: pale brown

Summarized from [9] with slight modifications.

**Table 3:** The constituents identified from the essential oil of *C. odorata*.

Class	Constituents	Plant parts	Reference
Monoterpenes	(*E*)-*β*-Ocimene	Leaf, fruit	[10–12]
	(*Z*)-*β*-Ocimene	Leaf, fruit	[10–12]
	1,8-Cineole	Leaf, flower, fruit	[10, 12–14]
	Bornyl acetate	Leaf	[11]
	Camphene	Leaf, flower	[11]
	Geraniol	Leaf, flower	[11]
	Geranyl acetate	Flower	[11, 13]
	Limonene	Leaf, flower, fruit	[10–14]
	Linalool	Leaf, flower	[10–13]
	Linalyl acetate	Leaf	[11]
	Myrcene	Leaf, fruit	[10–12]
	Neral	Flower	[15]
	Nerol	Flower	[14]
	Neryl acetate	Flower	[15]
	*p*-Cymene	Leaf, fruit	[11, 12]
	Plinol a	Flower	[16]
	Plinol d	Flower	[16]
	Sabinene	Leaf, fruit	[10–12]
	Terpinen-4-ol	Leaf, fruit	[10–12]
	Terpinolene	Leaf, fruit	[10–12]
	Thujanol	Fruit	[12]
	*trans*-Linalool oxide acetate	Flower	[15]
	*trans*-*β*-Ocimene	Flower	[13, 14]
	*α*-Phellandrene	Leaf, fruit	[10–12]
	*α*-Pinene	Leaf, flower, fruit	[10–14]
	*α*-Pyronene	Fruit	[16]
	*α*-Terpinene	Leaf, fruit	[10–12]
	*α*-Terpineol	Leaf, fruit	[10, 12–14]
	*α*-Thujene	Leaf, fruit	[11, 12]
	*β*-Myrcene	Flower	[13, 14]
	*β*-Phellandrene	Leaf	[11]
	*β*-Pinene	Leaf, flower, fruit	[10–14]
	*γ*-Terpinene	Leaf, fruit	[10–12]

Sesquiterpenes	(*E,E*)-Farnesal	Leaf	[11]
	(*E,E*)-Farnesol	Leaf, flower	[11]
	(*E,E*)-*α*-Farnesene	Flower	[11, 13–15]
	(*E,Z*)-Farnesal	Leaf	[11]
	(2*E*,2*Z*)-Farnesal	Flower	[15]
	(2*Z,*6*E*)-Farnesyl acetate	Flower	[15]
	1,10-diepi-Cubenol	Flower	[15]
	1*H*-Indole	Flower	[16]
	1-epi-Cubenol	Flower	[15]
	5-Indanol	Flower	[15]
	Aromadendrene	Leaf	[11]
	Bicycloelemene	Flower	[15]
	Bicyclogermacrene	Leaf	[10–12]
	Calamene	Flower	[13]
	Caryophyllene epoxide	Leaf	[10]
	Caryophyllene oxide	Leaf, flower	[11, 12, 14]
	Cedrol	Flower	[13, 14]
	Copaborneol	Flower	[15]
	Cyperene	Flower	[15]
	Germacrene D	Leaf, flower, fruit	[10–14]
	Globulol	Leaf	[11]
	Guaiol	Flower	[15]
	Isogermacrene D	Flower	[15]
	Jejunol	Flower	[15]
	Levoglucosenone	Flower	[16]
	Selina-4(15),5-diene	Flower	[15]
	Spathulenol	Leaf	[11]
	*t-*Cadinol	Leaf	[10, 12]
	*t*-Muurolol	Flower	[13, 14]
	*trans*-Nerolidol	Flower	[13, 14]
	Viridiflorol	Leaf	[11]
	Zonarene	Flower	[15]
	*α*-Amorphene	Leaf, flower	[10, 12]
	*α*-Bisabolol	Flower	[13, 14]
	*α*-Bulnesene	Leaf	[11]
	*α*-Cadinol	Leaf	[10, 12]
	*α*-Cedrene	Flower	[13]
	*α*-Copaene	Leaf, flower	[10–12]
	*α*-Cubebene	Leaf	[11]
	*α*-Gurjunene	Leaf	[11, 12]
	*α*-Humulene	Leaf, flower, fruit	[10–14]
	*α*-Muurolene	Leaf	[10, 12]
	*α*-Ylangene	Leaf, flower	[10, 12–14]
	*β*-Bourbonene	Leaf, flower	[11, 14, 15]
	*β*-Caryophyllene	Leaf, flower, fruit	[10–12]
	*β*-Copaene	Leaf	[12]
	*β*-Cubebene	Leaf, flower	[10–12, 14]
	*β*-Elemene	Leaf	[11, 12]
	*γ*-Cadinene	Leaf	[10, 12]
	*γ*-Muurolene	Flower, fruit	[13]
	*δ*-Cadinene	Leaf, flower	[10–12]
	*δ*-Cadinol	Flower	[13, 14]
	*δ*-Elemene	Leaf	[10, 12]
	*ε*-Cadinene	Flower	[13]
	*τ*-Cadinene	Flower	[13]
	*τ*-Cadinol	Flower	[13, 14]
	*τ*-Muurolene	Flower	[13]

Aliphatic compounds	(2*E*,6*E*)-Farnesyl acetate	Flower	[11, 13, 14, 17]
	(*E*)-Hex-2-enal	Leaf	[10, 12]
	(*E*)-Hex-2-enol	Leaf, flower	[10, 12]
	(*Z*)-Hex-3-enol	Leaf, flower	[10, 12]
	2-Hexenyl acetate	Flower	[13, 14]
	2-Methyl-3-buten-2-ol	Flower	[13, 14]
	3-Hexenyl acetate	Flower	[13, 14]
	3-Methyl-2-buten-1-ol	Flower	[13, 14]
	3-Methyl-2-buten-1-yl acetate (prenyl acetate)	Flower	[14, 17]
	Benzyl alcohol	Flower	[13, 14]
	Decane	Flower	[16]
	Diethyl 1,5-pentanedioate	Flower	[16]
	Dodecane	Flower	[16]
	Methyl 3-methylbutanoate	Flower	[16]
	Methyl caprylate	Flower	[16]
	*n*-Hexanol	Leaf, fruit	[10, 12]
	Heptanal	Flower	[15]
	Tetracosane	Flower	[15]
	Tricosane	Flower	[15]
	Undecane	Flower	[16]

Phenylpropanoids	(*E*)-Cinnamyl acetate	Flower	[11, 13]
	1,4-Dimethylbenzene	Flower	[14]
	1-Methoxy-1-propylbenzene	Flower	[16]
	1-Phenyl-2-propen-1-ol	Flower	[16]
	1-Phenylallyl acetate	Flower	[16]
	2-Methoxy-4-methylphenol	Flower	[14]
	2-Phenylethyl acetate	Flower	[13]
	3,4-Dimethoxytoluene	Flower	[14]
	3-Buten-2-ol benzoate	Flower	[14]
	3-Hexen-1-ol benzoate	Flower	[14]
	3-Methyl-2-buten-1-yl benzoate	Flower	[15]
	4-(2-Propenyl)-phenol	Flower	[14]
	4-Allyl-phenyl-acetate	Flower	[15]
	4-Methoxy benzaldehyde	Flower	[16]
	4-Methoxyphenyl acetate	Flower	[14]
	Anethol	Flower	[13, 14]
	Benzyl acetate	Flower	[11, 13, 14]
	Benzyl benzoate	Flower	[11, 13, 14]
	Benzyl salicylate	Flower	[11, 13, 14]
	Benzylaldehyde	Flower	[14]
	Benzyl-n-butyrate	Flower	[14]
	Butyl benzoate	Flower	[14]
	Cinnamyl alcohol	Flower	[14]
	Ethyl benzoate	Flower	[13, 14]
	Isoeugenol	Flower	[14]
	Methoxyphenol	Flower	[14]
	Methyl benzoate	Flower	[11, 13, 14]
	Methyl-2-methoxybenzoate	Flower	[14]
	Methyl-4-methoxybenzoate	Flower	[14]
	Methyleugenol	Flower	[13, 14]
	*p*-Cresyl methyl ether (*p*-methylanisole)	Flower	[13, 17, 18]
	*p-*Vinyl-guaiacol	Flower	[15]
	Vanillin	Flower	[15]
	Veratrole	Flower	[15]

Nitrogen-bearing compounds	Phenylacetonitrile	Flower	[14]
	2-Phenyl-1-nitroethane	Flower	[14]
	Methyl anthranilate	Flower	[14]

**Table 4:** The identified chemical constituents from different extracts of *C. odorata*.

Extracts	Family	Name of constituents	References
Methanolic extract of *C. odorata *seed	Quinoline alkaloids	(+)-Ushinsunine-*β*-*N*-oxide	[18]
		Cleistopholine	[18]
		Liriodenine	[18]
		(−)-Anonaine	[18]
		(+)-Nornuciferine	[18]
		(+)-*N-*Acetylnornuciferine	[18]
		(−)-Ushinsunine	[18]
		(−)-Norushinsunine	[18]
		(−)-Asimilobine	[18]
		(+)-Reticuline	[18]
		Lyscamine	[18]
		(−)-Anaxagoreine	[18]
	Phytosterols	Stigmasterol	[18]
		*β*-Sitosterol	[18]
	Phenylpropanoids	*N-trans*-Feruloyltyramine	[18]
		*trans*-Cinnamic acid	[18]

Chloroform extract of *C. odorata *stem bark	Quinoline alkaloids	Liriodenine	[19]
		Sampangine	[19]

Methanolic extract of *C. odorata * fruit	Guaipyridine alkaloids	Cananodine	[20]
	Cycloeudesmane sesquiterpenoids	Cryptomeridiol 11-*α*-L-rhamnoside	[20]
		*γ*-Eudesmol 11-*α*-L-rhamnoside	[20]
		*γ*-Eudesmol	[20]
	Quinoline alkaloids	Cleistopholine	[20]
		(+)-Ushinsunine-*β-N*-oxide	[20]
		Lyscamine	[20]
	Phenylpropanoids	*N-trans-*Feruloyltyramine	[20]

Acetone extract of *C. odorata *stems and leaves	Lactones	Isosiphonodine	[21]
		Canangone	[21]

Methanol extract of dried leaves of *C. odorata *	Megastigmane glycoside	Canangaionoside	[22]
		Breyniaionoside A	[22]
		Citroside A	[22]

Methanol extract of flower buds of *C. odorata *	Lignans	Canangalignan I	[8]
		Canangalignan II	[8]
		Canangaterpene I	[8]
	Terpenoids	Canangaterpene II	[8]
		Canangaterpene III	[8]
		Canangaterpene IV	[23]
		Canangaterpene V	[23]
		Canangaterpene VI	[23]
		(3R,3aR,8aS)-3-Isopropyl-8a-methyl-8-oxo-1,2,3,3a,6,7,8,8a-octahydroazulene-5-carbaldehyde	[8]

Methanol extract of leaves of *C. odorata var. fruticosa *	Monoterpene glucosides	Canangafruticoside A	[24]
		Canangafruticoside B	[24]
		Canangafruticoside C	[24]
		Canangafruticoside D	[24]
		Canangafruticoside E	[24]
	Ionone glucosides	Corchoionoside C	[24]
	Lignans	(+)-Syringaresinol 4-*O-β*-D-glucopyranoside	[24]

**Table 5:** The antimicrobial activities screening of different *C. odorata* extracts.

Plant part	Extracts	Pathogens tested	Screening assay	Reference
Bark	n/a	Gram-positive bacteria	Disc diffusion assay	[25]
		* Bacillus subtilis *		
		* Bacillus megaterium *		
		* Staphylococcus aureus *		
		* Sarcina lutea *		
		* Streptococcus-β-haemolyticus *		
		Gram-negative bacteria		
		* Escherichia coli *		
		* Pseudomonas aeruginosa *		
		* Shigella flexneri *		
		* Shigella shiga *		
		* Shigella boydii *		
		* Shigella dysenteriae *		
		* Shigella sonnei *		
		* Salmonella typhi *		
		* Klebsiella *		
		Fungi		
		* Aspergillus flavus *		
		* Aspergillus niger *		
		* Aspergillus versicolor *		
		* Candida albicans *		
	*n-*hexane	Gram-positive bacteria	Well diffusion assay	[26]
		* Propionibacterium acnes *		
		Fungi		
		* Candida albicans *		
	Ethyl acetate	Gram-positive bacteria		[26]
		* Propionibacterium acnes *		
		Fungi		
		* Candida albicans *		
	Ethanolic	Gram-positive bacteria		[26]
		* Propionibacterium acnes *		
		Fungi		
		* Candida albicans *		
	Ethanolic	Protozoan parasite	*In vitro* bioassay	[27]
	Cyclohexane	* Plasmodium falciparum *FcB1 strain		
	Methylene chloride			
	Methanolic			

Whole plant	Essential oils	Gram-positive bacteria	Disc diffusion assay	[28]
		Methicillin-resistant *Staphylococcus aureus *ATCC 700699		
		Gram-positive bacteria		[29]
		* Bacillus cereus *		
		* Bacillus subtilis *		
		* Bacillus megaterium *		
		* Bacillus polymyxa *		
		* Streptococcus-β-haemolyticus *		
		* Streptococcus aureus *		
		* Streptococcus lutea *		
		Gram-negative bacteria		
		* Escherichia coli *		
		* Shigella dysenteriae *		
		* Shigella flexneri *		
		* Shigella sonnei *		
		* Pseudomonas aeruginosa *		
		* Salmonella typhi B *		
		* Salmonella paratyphi A *		
		* Salmonella paratyphi B *		
		Fungi		[30]
		* Rhizopus oryzae *		
		* Aspergillus niger *		
		* Aspergillus fumigatus *		
		* Aspergillus krusli *		
		* Candida albicans *		
		* Saccharomyces cerevisiae *		
		Fungi		
		* Candida albicans* ATCC 48274		
		* Rhodotorula glutinis* ATCC 16740		
		* Schizosaccharomyces pombe* ATCC 60232		
		* Saccharomyces cerevisiae* ATCC 2365		
		* Yarrowia lipolytica* ATCC 16617		

Leaf	Methanolic	Gram-positive bacteria	Well diffusion assay	[31]
		* Staphylococcus aureus *		
		Gram-negative bacteria		
		* Salmonella typhi *		
		* Escherichia coli *		
		* Vibrio cholera *		
		Fungi		
		* Epidermophyton floccosum *		
		* Microsporum gypseum *		
		* Trichophyton mentagrophytes *		
		Protozoan parasite	*In vitro* bioassay	[27]
		* Plasmodium falciparum *FcB1 strain		
	Petroleum ether	Gram-positive bacteria	Well diffusion assay	[31]
		* Staphylococcus aureus *		
		Gram-negative bacteria		
		* Salmonella typhi *		
		* Escherichia coli *		
		* Vibrio cholera *		
		Fungi		
		* Epidermophyton floccosum *		
		* Microsporum gypseum *		
		* Trichophyton mentagrophytes *		
	Chloroform	Gram-positive bacteria	Well diffusion assay	[31]
		* Staphylococcus aureus *		
		Gram-negative bacteria		
		* Salmonella typhi *		
		* Escherichia coli *		
		* Vibrio cholera *		
		Fungi		
		* Epidermophyton floccosum *		
		* Microsporum gypseum *		
		* Trichophyton mentagrophytes *		
	Ethanolic	Protozoan parasite	*In vitro *bioassay	[27]
	Cyclohexane	* Plasmodium falciparum *FcB1 strain		
	Methylene chloride			

n/a: not available.

**Table 6:** Bioactivities of *C. odorata *essential oils and extracts.

Bioactivities	Part used	Type of extracts	Dosage/Results	Suggested constituents with respective activities	References
Antimicrobial (i) Antibacterial (ii) Antifungal (iii) Antiprotozoal	(i) Whole plant (ii) Bark (iii) Leaf	(i) Essential oil (ii) *n-*Hexane (iii) Ethyl acetate (iv) Ethanolic (v) Methanolic (vi) Cyclohexane (vii) Petroleum ether (viii) Chloroform	(i) Well diffusion assay: 100–400 *µ*g/well tested against variety of Gram-positive, Gram-negative bacteria and fungi	(i) Linalool (ii) Linalyl acetate (iii) Liriodenine (iv) *O-*Methylmoschatoline (v) 3,4-Dihydroxybenzoic acid (vi) Methyl eugenol (vii) n/a (viii) n/a	[26]
			(ii) Disc diffusion assay: 200–400 *μ*g/disc tested against variety of Gram-positive, Gram-negative bacteria and fungi		[28]
			(iii) 0.23 mg/mL (MIC_90%_) against *S. aureus *		[32]
			(iv) 12.5 ± 3.9 *µ*g/mL (IC_50_) against *P. falciparum* FcB1 strain		[33]

Antibiofilm	Flower	Essential oil	(i) 0.01% (v/v) showed 80% inhibition against biofilm for *S. aureus *ATCC 6538	(i) *cis*-Nerolidol (ii) *trans*-Nerolidol	[5]
			(ii) Inhibit adherence phase of both clinical strains of *S. aureus *and *K. pneumonia* (2 logs reduction)		

Antioxidant	(i) Bark (ii) Leaf (iii) Flower	(i) Ethyl acetate (ii) Methanolic (iii) Essential oil	(i) 79% DPPH inhibition tested at 50 ppm	(i) n/a	[26]
			(ii) 290.0 ± 13.1% of ferric reducing power at 0.5 *µ*g/mL	(ii) n/a	[34]
			(iii) 63.8 ± 0.45% of DPPH inhibition	(iii) n/a	[30]
			(iv) 75.5 ± 0.51% inhibition in the *β*-carotene bleaching test (v) DPPH radical scavenging activity (80.06 ± 0.02%)		

Insecticidal (i) *A. aegypti* (ii) *C. quinquefasciatus* (iii) *An. Dirus* (mosquitoes)	Flower	Essential oil	(i) Tested 1%, 5%, and 10% (w/v) on *A. aegypti*, *C. quinquefasciatus,* and *An. Dirus*, LC_50_ values of 9.77%, 8.82%, and 4.99%, respectively	(i) n/a	[35]
			(ii) 10% in soybean oil exhibited oviposition-deterrent and ovicidal activities	(ii) n/a	[36]
			(iii) 0.1 mg/mL showed larvicidal activity against *A. aegypti *	(iii) n/a	[37]
			(iv) LD_50_ at 52.96 ppm against immature stage of *A. aegypti *	(iv) n/a	[38]
(iv) *Musa domestica* (housefly)			(v) Prepared in ethyl alcohol, LT_50 _of 52.08 hours, and LC_50 _of 29.36% towards *Musa domestica *	(v) n/a	[39]
(v) *R. speratus* (termite)			(vi) 2 mg/filter showed 18.0 ± 5.8% and 94.0 ± 4.0% mortalities after 2 and 7 days of exposure	(vi) n/a	[40]
(vi) *S. zeamais *(agriculture pest)			(vii) LD_50 _value of 33.14 *μ*g/adult	(vii) Linalool	[41]
			(viii) LD_50_ value of 14.77 mg/L (vapour phase toxicity bioassay)		

Insect repellent (i) *A. aegypti* (ii) *C. quinquefasciatus* (iii) *An. dirus *(mosquitoes) (iv) *T. castaneum *(beetle)	(i) Flower (ii) Leaf	Essential oil	(i) Prepared in soybean oil, ED_50_ of 0.045, 2.149, and <0.003 mg/cm^2^against *A. aegypti, A. dirus,*and *C. quinquefasciatus,*respectively	(i) Linalool	[42]
			(ii) Protection time towards *A. aegypti*, *A. dirus,* and *C. quinquefasciatus* (8.4, 24.0, and 60.0 minutes, resp.)		
			(iii) Prepared in ethyl alcohol, protection time against *A. aegypti,* and *C. quinquefasciatus* (86.67 ± 10.40 and 126.0 ± 15.77 minutes) at 0.33 *µ*L/cm^2^	(ii) n/a	[43]
			(iv) Strongest repellent effect at 5 *μ*L/g of oats	(iii) n/a	[3]
			(v) 98% repellency after 2 and 4 hours exposure		

Antimelanogenesis	(i) Flower bud	Methanolic	(i) Inhibition on melanin production in B16 melanoma 4A5 cells	(i) Canangaterpenes I (ii) (3R,3aR,8aS)-3-Isopropyl-8a-methyl-8-oxo-1,2,3,3a,6,7,8,8a-octahydroazulene-5-carbaldehyde	[8]
			(ii) Terpenoid derivatives, canangaterpenes I (IC_50 _= 3.6 *µ*M), and (3R,3aR,8aS)-3-isopropyl-8a-methyl-8-oxo-1,2,3,3a,6,7,8,8a-octahydroazulene-5-carbaldehyde (IC_50_ = 2.5 *µ*M)		
	(ii) Seed		(iii) Inhibition on tyrosinase protein expression in mouse B16 melanoma cells	(iii) *N-trans*-Feruloyltyramine	[18]

Anti-inflammatory	(i) n/a (ii) Leaf (iii) Fruit	(i) Essential oil (ii) Methanolic (iii) Ethanolic	(i) Strong lipoxygenase inhibitory effect (~80%) at 0.5 *μ*g/mL	(i) Linalool	[6]
			(ii) Inhibition on nitric oxide release in RAW264.7 (97.9 ± 14.6%) at 50 *µ*g/mL	(ii) Linalyl acetate	[34]
			(iii) In carrageenan induced paw edema model, paw volume inhibition of 62.9% at 100 mg/kg	(iii) n/a	[44]

Sedative, relaxing, and harmonizing effect	n/a	Essential oil	(i) Reduced systolic and diastolic BP through sniffing	(i) n/a	[45]
			(ii) Decreased pulse rate and stress level		
			(iii) Increased alertness		
			(iv) Transdermal administration resulted decrease in both physiological and behavioural level	(ii) n/a	[46]

Effect on mood and cognitive performance	n/a	Essential oil	Reduced alertness mood and calmness but without increased cognitive performance	n/a	[47]

Spermatotoxic	Root bark	Ethanolic	(i) Immobilized rat's sperm within seconds	(i) A 52 kd protein	[48]
			(ii) 50 mg/100 g body weight/day reduced sperm motility	(ii) n/a	[49]
			(iii) 100 mg/100 g body weight/day caused 94% abnormal sperm morphology		

Antihyperglycemic	(i) Leaf and stem (ii) Flower buds	(i) Dichloromethane (ii) Methanolic	(i) Alpha-amylase inhibitory effect with 22.6 ± 1.3% (leaf) and 25.3 ± 3.3% (stem) inhibition at 7.8 *μ*g/mL	(i) n/a	[7]
			(ii) Aldose reductase inhibitory effect, IC_50_ at 1.2, 1.5, and 0.8 *μ*M by canangaterpene I, (*E*)-[(1R,3R,5S,6S,8S)-6-hydroxy-1,3-dimethoxy-2-oxaspiro[4,5]decan-8-yl]methyl] caffeate, and canangafruiticoside E respectively	(ii) Canangaterpene I (iii) (*E*)-[(1R,3R,5S,6S,8S)-6-Hydroxy-1,3-dimethoxy-2-oxaspiro[4,5]decan-8-yl]methyl] caffeate (iv) Canangafruiticoside E	[23]

IC_50_: half maximal inhibitory concentration.

LD_50_: median lethal dose.

LT_50_: median lethal time.

ED_50_: median effective dose.

n/a: not available.
